# A Survey on Clinical Governance Awareness Among Clinical Staff: A Cross-Sectional Study

**DOI:** 10.5539/gjhs.v6n6p37

**Published:** 2014-06-24

**Authors:** Hamid Ravaghi, Rahim Khodayari Zarnaq, Amin Adel, Mahnaz Badpa, Moein Adel, Nazanin Abolhassani

**Affiliations:** 1 Health Management and Economics Research Center, School of Health Management and Information Sciences, Iran University of Medical Sciences, Tehran, Iran; 2 Department of Health Management and Economics, School of Public Health, Tehran University of Medical Sciences, Tehran, Iran; 3 Department of Health Services Management, School of Public Health, Mashhad University of Medical Sciences, Mashhad, Iran; 4 Department of Medical Nanotechnology, School of Advance Technologies in Medicine, Tehran University of Medical Sciences, Tehran, Iran

**Keywords:** clinical governance, awareness, clinical staff, hospital

## Abstract

**Objective:**

Clinical Governance (CG) program has been raised in Iran in order to improve the quality of clinical care. The purpose of this study is to investigate the awareness of clinical governance program among clinical staff working in selected teaching hospitals in Tehran, Iran.

**Methods:**

To investigate the CG awareness, a cross-sectional survey was conducted among 345 clinical staff working in 20 selected public hospitals in Tehran. Data were gathered using the standardized clinical governance awareness questionnaire. Descriptive statistics were used to analyze the data.

**Result:**

The results showed that the level of staff awareness about the concept of CG was low. They perceived continuous quality improvement, responsibility, medical errors reduction and patient safety as the main concepts of the CG framework. Reaching agreement of standards concepts among staff and positive changes in attitudes were considered as two most observed changes. The main perceived barriers to the implementation of clinical governance included lack of proper management and leadership, lack of full support, inappropriate organizational culture, lack of knowledge, poor communication system and insufficient training.

**Conclusions:**

The concepts and goals of clinical governance have not been effectively conveyed to the staff and despite its implementation in the hospitals, there has been low clinical governance awareness among the staff. Clinical Governance must be implemented through comprehensive management support and participation of all staff and health professionals at both hospital and policy making level.

## 1. Introduction

In order to respond to the increasing demand for clinical effectiveness, efficiency and value for money in health sector, Clinical Governance (CG) framework has been recently emerged in some countries. Although, there are different competing definitions of CG, perhaps the classic definition is provided by Scally and Donaldson as “a system through which [health] organizations are accountable for continuously improving the quality of their services and safeguarding high standards of care by creating an environment in which excellence in clinical care will flourish” (1998, p. 62).

CG considers both responsibilities to maintain the current level of care and the improvement of the quality of the future care simultaneously. Indeed, it provides a unique and comprehensive strategy of continuous quality improvement as a systematic model (Haxby et al., 2010; Staniszewska et al., 2008). It aims to unify and integrate all quality improvement activities addressing both clinical and non-clinical services. This comprehensive approach merges all patient care activities into a single strategy leading to organizational integration, coordination, cooperation and communication between departments (Greenfield et al., 2011; Travaglia et al., 2011; Tuan, 2012).

The implementation of CG requires the establishment of a culture which encourages health professionals to improve their performance and such a culture promotes continuous learning and recognizes it as the key of success for quality improvement (Adams & Janet, 2003). Learning, training and development of personnel knowledge are integrated components of CG (Dixon-Woods et al., 2012). Studies showed that the main issues and challenges should be taken into account when implementing the program. The issues such as personnel knowledge, financial resources, required infra structure, proper information systems, identification of functional indices and effectiveness considerations, networking, difficulties in the standardization, and staff encouragement to be involved in the program have been identified (McColl & Roland, 2000; Rosen, 2000).

The World Health Organization recommends its member states to implement CG (Rashidian, 2012). Iran ministry of health has promoted CG as a model to improve quality of care in all hospitals since 2009 (Ravaghi et al., 2014), consisting of seven inter-locking components, namely the seven Pillars model (Nicholls et al., 2000). The components are clinical effectiveness, clinical audit, risk management, patient and public involvement, education and training, staff management and use of information. Factors including systems awareness, leadership, teamwork, ownership, and communication compose the foundation of the model.

Initial evaluations show that Iran healthcare system has faced some challenges regarding the implementation of CG; such as lack of adequate physicians’ participation in the program, inadequate authority of managers, educational and quality related issues as well as poor knowledge (Rashidian, 2012; Heyrani et al,. 2012). Since educational programs for CG have been initiated in most hospitals throughout the country, level of staff awareness about CG concepts and its goals is of importance. In addition, the main challenges of the implementation of CG are related to the staff (educational challenges, lack of enough participation and poor knowledge) which necessitates the study of staff awareness about CG. The result of the study may provide information for next steps of the implementation of CG in Universities of medical sciences, hospitals and healthcare centers in Iran. This study, therefore, aims to investigate the awareness of CG among clinical staff working in selected teaching hospitals in Tehran, Iran.

## 2. Methods

To investigate the CG awareness, a cross-sectional survey was conducted among 345 clinical staff working in 20 selected public hospitals in Tehran. There are 40 teaching hospitals in Tehran and half of them were randomly selected. A random sampling, proportional to the number of clinical staff in each hospital, was applied to select study participants. A total number of 403 people participated in the study; 345 of them (85.6%) returned completed questionnaires. Data were gathered using the clinical governance awareness questionnaire, a standard questionnaire developed by Mc Sherry and Pearce (2011). The questionnaire has 4 parts. The first part (5questions) asks about demographic characteristics of respondents, the second part (15 questions) is about the awareness of CG concepts. The third part (6questions) asks about staff awareness of components of CG and finally, the forth part is about barriers to implement CG. Face validity of the questionnaire was established by asking the faculty members and staff working in the Clinical Governance department of the Ministry of Health. Reliability, assessed by Cronbach’s alpha, was 0.81. Descriptive statistics were applied to analyze the data using SPSS 18 and Microsoft Excel 2010. Ethics approval was obtained from the local ethics committee (No. 16241).

## 3. Results

There were 345 clinical staff participating in the study; 66.8% were female and 31.8%were male. The mean age and years of experience across the respondents were 36 and 12.5, respectively. Most of them (78%) were staff nurses and nursing supervisors while the rest were physicians and clinical administrators.

The study findings indicate that the level of staff awareness of the concepts of CG was low (Table 1).

**Table 1:** Staff’s views on CG

Factor	Mean±SD	Highest attainable score
CG role in improving patient care situation	2.12±0.12	
CG as roles and responsibilities of staff	1.83±0.64	
Benefit of CG in professional practice	1.90±0.56	
CG effect on patient care	1.93±0.66	
CG effect on changing clinical practice at individual, team and organizational level	1.94±0.58	
CG effect on positive change in the organizational culture	2.02±0.7	
Giving staff and physicians sufficient support and encouragement to implement CG	2.72±0.97	5
Getting sufficient support of management within clinical area to implement CG	2.73±1.02	
Receiving adequate information about CG and its impact on quality improvement	2.41±1.08	
Having basic knowledge about CG and its related systems and processes	2.41±0.95	
Being confident to engage with the CG framework	2.55±0.75	
Total	2.23±0.41	

Table 1 shows that the average level of staff awareness of CG was low (2.23 out of 5). Mean while 97% of staff mentioned that they had received introductory training in CG.

The participants perceived CG concept in different terms. Table 2 shows the staff perception of the CG concept as below:

**Table 2:** Staff perceptions of the CG concept

Staff perceptions	Number	Percentage
Continuous quality improvement	109	31.6
Responsibility	100	28.9
Medical errors reduction	43	12.5
Patient safety	41	11.8
Another method for documentation	32	9.4
Continuous monitoring	20	5.8
Total	345	100

As seen in Table 2, more than 50% of staff believed that CG signifies the continuous quality improvement and responsibility. Others pointed to concepts such as patient safety and medical errors reduction.

Among 345 participants, 271 staff (78%) reported a range of changes in practice that was related from individuals, teams or organization engaging in the CG framework. Reaching agreement of standards concepts and positive changes in attitudes were considered as two most observed changes. Table 3 shows types of perceived changes in practice.

**Table 3:** Types of changes caused by the implementation of CG

Type of change	Number	Percentage
Agreed and shared understanding of standards	62	22.8
Changes in attitudes	54	20
Reduction of medical errors	51	18.7
Modifying some processes	47	17.4
Understanding patient rights	39	14.4
Other changes	18	6.7
Total	271	100

As shown in Table 4, staff mentioned six factors as greatest barriers affecting staff engagement with the CG framework. Lack of proper management and leadership and lack of full support were two most perceived barriers.

**Table 4:** Greatest barriers affecting staff engagement with the CG framework

Barriers	Number	Percentage
Lack of proper management and leadership	96	27.8
Lack of full support	90	26
Inappropriate organizational culture	54	15.7
Lack of knowledge	41	11.8
Poor communication system	33	9.5
Insufficient training	32	9.2
Total	345	100

## 4. Discussion

This study aims to investigate the awareness of CG among clinical staff working in selected teaching hospitals in Tehran. According to the results, staff awareness of the concept of CG was low. They perceived it differently and mainly emphasized the six concepts including continuous quality improvement, responsibility, medical errors reduction, patient safety, another method for documentation and continuous monitoring. More than half of the respondents emphasized the concept of continuous quality improvement and responsibility. The continuous quality improvement was strongly emphasized by the nurses, while responsibility was highlighted mostly by the managers, supervisors and physicians. Such emphasis may be due to the nature of their jobs. Mohaghegh and Ravaghi (2013) also found similar results in a qualitative study conducted in selected Iranian hospitals. They also considered CG as an efficiency tool while it has not been mentioned in the current study.

The concepts including medical errors reduction and patient safety along with patient and community involvement, teaching and leading, use of information, clinical effectiveness, clinical audit and human resource management are principles of CG found by other studies (Hadizadeh et al., 2007; Linda et al, 2007). In this study, staff had difficulties to differentiate between concepts of CG and its principles as it was proposed in the questionnaire. It may be due to inadequate understanding and familiarity of the staff with CG as a new concept.

A majority of staff have believed that in order to achieve the objectives of CG certain programs must be implemented, such as risk management, performance management and information management, in consistence with findings of other studies (Firth-Cozens, 1999; Goodman, 1998). Another study also concluded that the CG program has led to medical errors reduction, more patient participation and increased use of information systems and more participation of staff in training programs (Khodayari Zarnaq et al., 2012).

Additionally, staff has mentioned greatest barriers to implementing CG as follows: lack of proper management and leadership, lack of full support, inappropriate organizational culture, lack of knowledge, poor communication system and insufficient training. McSherry and Pearce (2011) has divided barriers into 4 groups: individual internal barriers (including lack of knowledge and confidence sense of ownership, resistance against change and information); Organizational internal barriers (including inappropriate culture, poor management and leadership and lack of information); Individual external barriers (including lack of support, resources and time); and organizational external barriers (including political pressures, increased demand for irrational services, public expectations and lack of resources). It seems that the clinical staff working in hospitals grumbled mainly about organizational internal barriers.

Ravaghi et al. (2013) also stated that CG will fail unless adequate resources, essential structures, clinical staff ’s support from CG, stability of the program, resolving legal challenges and integrating different quality programs in a unique comprehensive program are provided. Similarly, Mohaghegh and Ravaghi (2013) in their study also have mentioned the shortage of manpower and budget, poor management system, inappropriate organizational structure and culture as main barriers to implement CG. In a study conducted by Karimi et al. (2012), greatest barriers to implementing CG were highlighted, consisting of staff shortage, lack of support and commitment from manager, poor training, inadequate motivational factors and inappropriate organizational culture. The staff expected more support from hospital senior managers, and at a higher level, top managers of universities of medical sciences and the ministry of health.

## 5. Conclusion

The concepts and goals of clinical governance have not been effectively conveyed to the staff and despite its implementation in the hospitals, there has been low clinical governance awareness among the staff. Therefore, providing more effective relevant training of the concept and components of CG is of great importance. Furthermore, in order to better CG implementation, managers should take the perceived barriers into account at both hospital and policy making levels. Clinical Governance must be implemented through comprehensive management support and participation of all staff and health professionals. Moreover, due to the inadequacy of empirical evidence, conducting studies to examine the different aspects of CG process and its effect is crucial.
